# The genome sequence of the European conger eel, *Conger conger *(Linnaeus, 1758)

**DOI:** 10.12688/wellcomeopenres.23052.1

**Published:** 2024-09-18

**Authors:** Patrick Adkins, Rachel Brittain, Joanna Harley, Vengamanaidu Modepali, Marine Biological Association Genome Acquisition Lab, Darwin Tree of Life Barcoding collective, Wellcome Sanger Institute Tree of Life Management, Samples and Laboratory team, Wellcome Sanger Institute Scientific Operations: Sequencing Operations, Wellcome Sanger Institute Tree of Life Core Informatics team, Tree of Life Core Informatics collective, Darwin Tree of Life Consortium

**Affiliations:** 1 The Marine Biological Association, Plymouth, England, UK

**Keywords:** Conger conger, European conger, genome sequence, chromosomal, Anguilliformes

## Abstract

We present a genome assembly from an individual *Conger conger* (the European conger eel; Chordata; Actinopteri; Anguilliformes; Congridae). The genome sequence spans 1,136.40 megabases. Most of the assembly is scaffolded into 19 chromosomal pseudomolecules. The mitochondrial genome has also been assembled and is 18.86 kilobases in length.

## Species taxonomy

Eukaryota; Opisthokonta; Metazoa; Eumetazoa; Bilateria; Deuterostomia; Chordata; Craniata; Vertebrata; Gnathostomata; Teleostomi; Euteleostomi; Actinopterygii; Actinopteri; Neopterygii; Teleostei; Elopocephalai; Elopocephala; Elopomorpha; Anguilliformes; Congridae; Congrinae; Conger; *Conger conger* (Linnaeus, 1758) (NCBI:txid82655).

## Background

The European Conger Eel *Conger conger* (Linnaeus, 1758) is the largest eel species found in Europe, distributed across the North-East Atlantic, Mediterranean, and western Black Sea ( Whitehead, 1985). Conger eels are strictly marine benthic fishes that live on rocky and sandy bottoms to 500 depth, although they travel much deeper to spawn ( Bauchot & Blache, 1980). Conger Eels are carnivores and mainly feed on bottom-living fishes, crustaceans, and cephalopods at night ( Cau & Manconi, 1984; Levy *et al.*, 1988; Saldanha *et al.*, 1995). Conger eels are the largest of the family Congridae with records existing of specimens of over 2.7 m and weighing 65 kg ( Wheeler, 1985), although such large fishes are uncommon ( Fannon *et al.*, 1990). Male conger eels are reported to be smaller than females ( Cau & Manconi, 1983). The European conger eel reaches sexual maturity at 5–15 years old and spawns terminally in deep waters during summer ( Hayward & Ryland, 2017). Female Conger eels have a semelparous reproductive strategy, reproducing only once and dying after releasing several million eggs. Spawning grounds for conger eels have been identified in the Sardinian Channel, between Gibraltar and the Azores, and near the Azores archipelago at depths of up to 4000 m ( Correia *et al.*, 2002; Correia *et al.*, 2009; Correia *et al.*, 2011; Correia *et al.*, 2012). Conger eel larvae are highly dispersive, and their development lasts for about 6–9 months before they metamorphose into juvenile eels ( Correia *et al.*, 2011).

Conger eels are important commercial fishing species of the North-East Atlantic ( Figueiredo *et al.*, 1996). Despite being a geographically widespread species and a valuable fisheries resource, relatively little is known about the reproductive biology, ecology, and migratory behaviour of Conger eels. The genome resource for the Conger eel adds valuable information to understanding their biology and population genetics, which could help in their conservation and management.

## Genome sequence report

**Figure 1.**
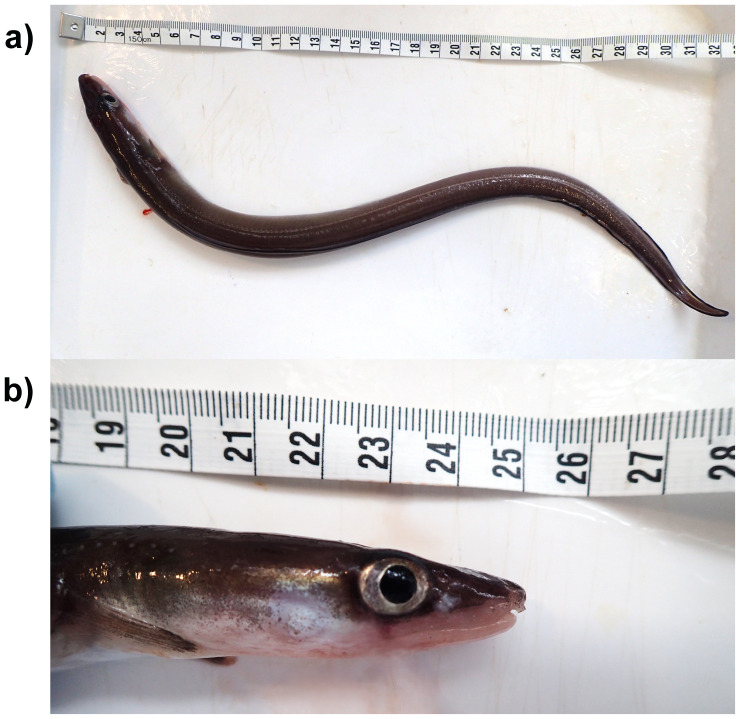
Photograph of the *Conger conger* (fConCon1) specimen used for genome sequencing.

The genome of a juvenile *Conger conger* ( Figure 1) was sequenced using Pacific Biosciences single-molecule HiFi long reads, generating a total of 17.62 Gb (gigabases) from 1.46 million reads, providing approximately 36-fold coverage. Primary assembly contigs were scaffolded with chromosome conformation Hi-C data, which produced 253.94 Gbp from 1,681.70 million reads, yielding an approximate coverage of 223-fold. Specimen and sequencing information is summarised in Table 1.

**Table 1.** Specimen and sequencing data for *Conger conger*.

Project information			
**Study title**	Conger conger (European conger)		
**Umbrella BioProject**	PRJEB65259		
**Species**	*Conger conger*		
**BioSample**	SAMEA12219431		
**NCBI taxonomy ID**	82655		
Specimen information			
**Technology**	**ToLID**	**BioSample accession**	**Organism part**
**PacBio long read sequencing**	fConCon1	SAMEA12219622	gill
**Hi-C sequencing**	fConCon1	SAMEA12219622	gill
**RNA sequencing**	fConCon1	SAMEA12219616	muscle
Sequencing information			
**Platform**	**Run accession**	**Read count**	**Base count (Gb)**
**Hi-C Illumina NovaSeq 6000**	ERR11872601	1.68e+09	253.94
**PacBio Sequel IIe**	ERR11867230	2.08e+06	20.78
**PacBio Sequel IIe**	ERR11867231	1.46e+06	17.62
**RNA Illumina NovaSeq 6000**	ERR12245589	8.38e+07	12.65

**Figure 2.**
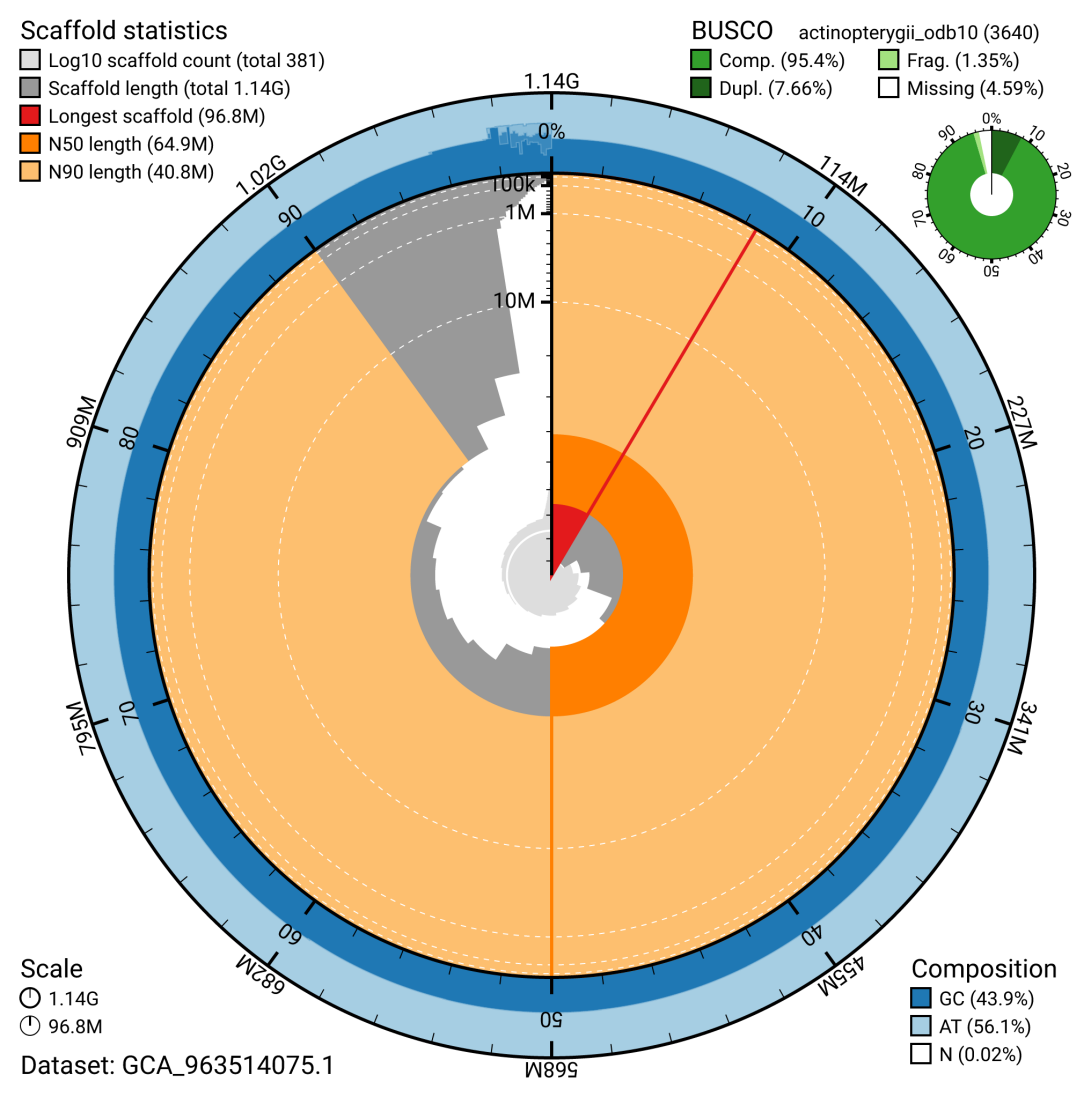
Genome assembly of *Conger conger*, fConCon1.1: metrics. The BlobToolKit snail plot shows N50 metrics and BUSCO gene completeness. The main plot is divided into 1,000 size-ordered bins around the circumference with each bin representing 0.1% of the 1,136,421,744 bp assembly. The distribution of scaffold lengths is shown in dark grey with the plot radius scaled to the longest scaffold present in the assembly (96,783,005 bp, shown in red). Orange and pale-orange arcs show the N50 and N90 scaffold lengths (64,882,367 and 40,826,945 bp), respectively. The pale grey spiral shows the cumulative scaffold count on a log scale with white scale lines showing successive orders of magnitude. The blue and pale-blue area around the outside of the plot shows the distribution of GC, AT and N percentages in the same bins as the inner plot. A summary of complete, fragmented, duplicated and missing BUSCO genes in the actinopterygii_odb10 set is shown in the top right. An interactive version of this figure is available at https://blobtoolkit.genomehubs.org/view/GCA_963514075.1/dataset/GCA_963514075.1/snail.

**Figure 3.**
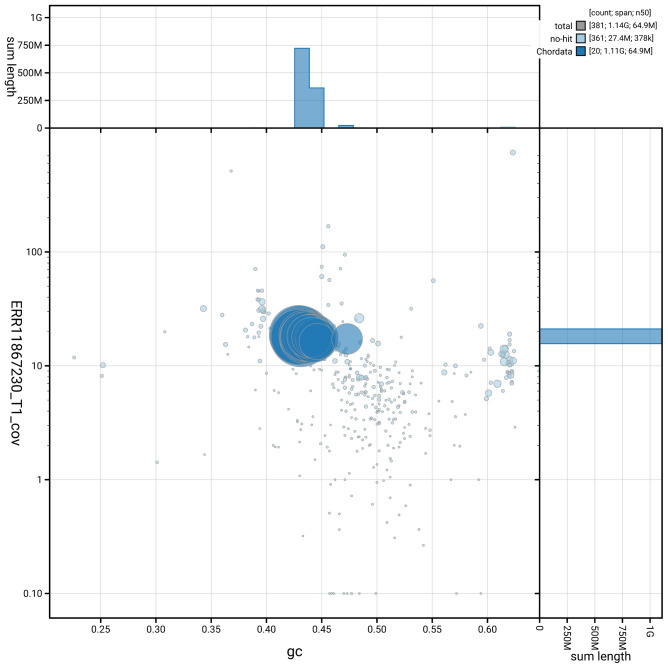
Genome assembly of *Conger conger*, fConCon1.1: BlobToolKit GC-coverage plot. Sequences are coloured by phylum. Circles are sized in proportion to sequence length. Histograms show the distribution of sequence length sum along each axis. An interactive version of this figure is available at https://blobtoolkit.genomehubs.org/view/GCA_963514075.1/dataset/GCA_963514075.1/blob.

**Figure 4.**
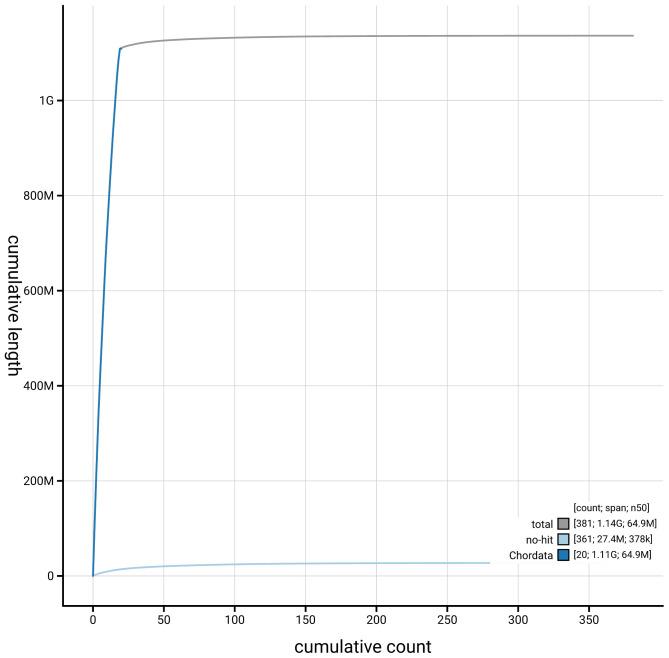
Genome assembly of *Conger conger* fConCon1.1: BlobToolKit cumulative sequence plot. The grey line shows cumulative length for all sequences. Coloured lines show cumulative lengths of sequences assigned to each phylum using the buscogenes taxrule. An interactive version of this figure is available at https://blobtoolkit.genomehubs.org/view/GCA_963514075.1/dataset/GCA_963514075.1/cumulative.

**Figure 5.**
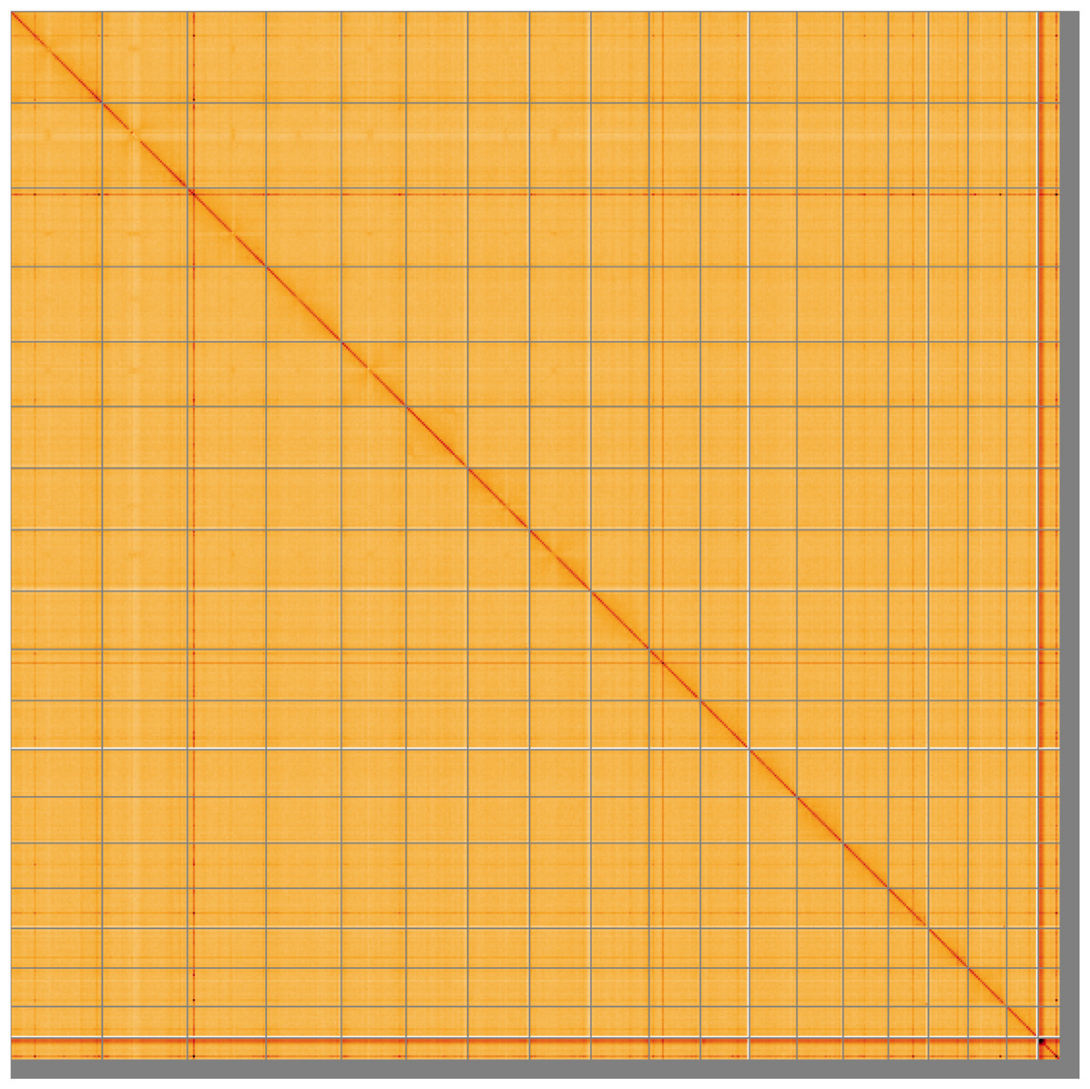
Genome assembly of *Conger conger* fConCon1.1: Hi-C contact map of the fConCon1.1 assembly, visualised using HiGlass. Chromosomes are shown in order of size from left to right and top to bottom. An interactive version of this figure may be viewed at https://genome-note-higlass.tol.sanger.ac.uk/l/?d=Wd1d4hWJSj-yRmTB5eV_PA.

Manual assembly curation corrected 23 missing joins or mis-joins, reducing scaffold number by 4.99%, and increasing the scaffold N50 by 37.08%. The final assembly has a total length of 1,136.40 Mb in 380 sequence scaffolds, with 1,009 gaps, and a scaffold N50 of 64.9 Mb ( Table 2). The snail plot in Figure 2 provides a summary of the assembly statistics, while the distribution of assembly scaffolds on GC proportion and coverage is shown in Figure 3. The cumulative assembly plot in Figure 4 shows curves for subsets of scaffolds assigned to different phyla. Most (97.58%) of the assembly sequence was assigned to 19 chromosomal-level scaffolds. Chromosome-scale scaffolds confirmed by the Hi-C data are named in order of size ( Figure 5; Table 3). While not fully phased, the assembly deposited is of one haplotype. Contigs corresponding to the second haplotype have also been deposited. The mitochondrial genome was also assembled and can be found as a contig within the multifasta file of the genome submission.

**Table 2.** Genome assembly data for *Conger conger*, fConCon1.1.

Genome assembly		
Assembly name	fConCon1.1	
Assembly accession	GCA_963514075.1	
*Accession of alternate haplotype*	*GCA_963514125.1*	
Span (Mb)	1,136.40	
Number of contigs	1,390	
Contig N50 length (Mb)	3.1	
Number of scaffolds	380	
Scaffold N50 length (Mb)	64.9	
Longest scaffold (Mb)	96.78	
Assembly metrics *		*Benchmark*
Consensus quality (QV)	56.1	*≥ 50*
*k*-mer completeness	99.99%	*≥ 95%*
BUSCO **	C:94.9%[S:87.4%,D:7.5%], F:1.8%,M:3.3%,n:3640	*C ≥ 95%*
Percentage of assembly mapped to chromosomes	97.58%	*≥ 95%*
Sex chromosomes	Not identified	*localised homologous pairs*
Organelles	Mitochondrial genome: 18.86 kb	*complete single alleles*

* Assembly metric benchmarks are adapted from column VGP-2020 of “Table 1: Proposed standards and metrics for defining genome assembly quality” from Rhie *et al.* (2021).

** BUSCO scores based on the actinopterygii_odb10 BUSCO set using version 5.4.3. C = complete [S = single copy, D = duplicated], F = fragmented, M = missing, n = number of orthologues in comparison. A full set of BUSCO scores is available at https://blobtoolkit.genomehubs.org/view/Conger_conger/dataset/GCA_963514075.1/busco.

**Table 3.** Chromosomal pseudomolecules in the genome assembly of *Conger conger*, fConCon1.

INSDC accession	Name	Length (Mb)	GC%
OY741314.1	1	96.78	43.0
OY741315.1	2	89.85	43.0
OY741316.1	3	83.29	43.0
OY741317.1	4	79.47	43.0
OY741318.1	5	68.37	43.5
OY741319.1	6	65.24	43.5
OY741320.1	7	65.18	43.5
OY741321.1	8	64.88	44.0
OY741322.1	9	61.47	43.5
OY741323.1	10	54.23	44.5
OY741324.1	11	51.85	44.0
OY741325.1	12	50.02	44.0
OY741326.1	13	48.9	44.0
OY741327.1	14	47.67	44.0
OY741328.1	15	42.41	44.5
OY741329.1	16	41.91	44.5
OY741330.1	17	40.83	44.0
OY741331.1	18	33.25	44.5
OY741332.1	19	23.41	47.5
OY741333.1	MT	0.02	37.0

The estimated Quality Value (QV) of the final assembly is 56.1 with *k*-mer completeness of 99.99%, and the assembly has a BUSCO v5.4.3 completeness of 94.9% (single = 87.4%, duplicated = 7.5%), using the actinopterygii_odb10 reference set ( *n* = 3,640).

Metadata for specimens, BOLD barcode results, spectra estimates, sequencing runs, contaminants and pre-curation assembly statistics are given at https://links.tol.sanger.ac.uk/species/82655.

## Methods

### Sample acquisition and barcoding

A juvenile *Conger conger* specimen (specimen ID MBA-210527-004A, ToLID fConCon1) was collected from Middle Ground, English Channel, UK (latitude 50.24, longitude –4.18) on 2021-05-27. The specimen was taken from its habitat Broken shell and muddy sand using an Agassiz trawl deployed from RV Sepia. The specimen was collected by Patrick Adkins and Joanna Harley (Marine Biological Association) and identified by Rachel Brittain (Marine Biological Association) based on gross morphology. The fish was first anesthetised and then overdosed using Aquased (2-phenoxyethanol). Destruction of the brain was used as a secondary method to ensure the animal was deceased before tissue sampling took place as in accordance with Schedule 1 methodology under the home office licence. Samples taken from the animal were preserved on dry ice.

The initial identification was verified by an additional DNA barcoding process according to the framework developed by Twyford *et al*. (2024). A small sample was dissected from the specimens and stored in ethanol, while the remaining parts of the specimen were shipped on dry ice to the Wellcome Sanger Institute (WSI). The tissue was lysed, the COI marker region was amplified by PCR, and amplicons were sequenced and compared to the BOLD database, confirming the species identification ( Crowley *et al.*, 2023). Following whole genome sequence generation, the relevant DNA barcode region was also used alongside the initial barcoding data for sample tracking at the WSI ( Twyford *et al.*, 2024). The standard operating procedures for Darwin Tree of Life barcoding have been deposited on protocols.io ( Beasley *et al.*, 2023).

### Nucleic acid extraction

The workflow for high molecular weight (HMW) DNA extraction at the WSI Tree of Life Core Laboratory includes a sequence of core procedures: sample preparation and homogenisation, DNA extraction, fragmentation and purification. Detailed protocols are available on protocols.io ( Denton *et al.*, 2023b).

The fConCon1 sample was weighed and dissected on dry ice ( Jay *et al.*, 2023). Tissue from the gill was homogenised using a PowerMasher II tissue disruptor ( Denton *et al.*, 2023a). HMW DNA was extracted using the Automated MagAttract v1 protocol ( Sheerin *et al.*, 2023). DNA was sheared into an average fragment size of 12–20 kb in a Megaruptor 3 system ( Todorovic *et al.*, 2023). Sheared DNA was purified by solid-phase reversible immobilisation, using AMPure PB beads to eliminate shorter fragments and concentrate the DNA ( Strickland *et al.*, 2023). The concentration of the sheared and purified DNA was assessed using a Nanodrop spectrophotometer and a Qubit Fluorometer using the Qubit dsDNA High Sensitivity Assay kit. The fragment size distribution was evaluated by running the sample on the FemtoPulse system.

RNA was extracted from muscle tissue of fConCon1 in the Tree of Life Laboratory at the WSI using the RNA Extraction: Automated MagMax™ *mir*Vana protocol ( do Amaral *et al.*, 2023). The RNA concentration was assessed using a Nanodrop spectrophotometer and a Qubit Fluorometer using the Qubit RNA Broad-Range Assay kit. Analysis of the integrity of the RNA was done using the Agilent RNA 6000 Pico Kit and Eukaryotic Total RNA assay.

### Library preparation and sequencing

Pacific Biosciences HiFi circular consensus DNA sequencing libraries were constructed according to the manufacturers’ instructions. Poly(A) RNA-Seq libraries were constructed using the NEB Ultra II RNA Library Prep kit. DNA and RNA sequencing was performed by the Scientific Operations core at the WSI on Pacific Biosciences Sequel IIe (HiFi) and Illumina NovaSeq 6000 (RNA-Seq) instruments.

Hi-C data were generated from frozen gill tissue of the fConCon11 sample, using the Arima-HiC v2 kit. The tissue was fixed with a TC buffer containing formaldehyde, resulting in crosslinked DNA. The crosslinked DNA was digested with a restriction enzyme master mix. The resulting 5’-overhangs were filled in and labelled with a biotinylated nucleotide. The biotinylated DNA was then fragmented, enriched, barcoded, and amplified using the NEBNext Ultra II DNA Library Prep Kit. Hi-C sequencing was performed on an Illumina NovaSeq 6000 instrument, using paired-end sequencing with a read length of 150 bp.

### Genome assembly, curation and evaluation

***Assembly***

The HiFi reads were first assembled using Hifiasm ( Cheng *et al.*, 2021) with the --primary option. Haplotypic duplications were identified and removed using purge_dups ( Guan *et al.*, 2020). The Hi-C reads were mapped to the primary contigs using bwa-mem2 ( Vasimuddin *et al.*, 2019). The contigs were further scaffolded using the provided Hi-C data ( Rao *et al.*, 2014) in YaHS ( Zhou *et al.*, 2023) using the --break option. The scaffolded assemblies were evaluated using Gfastats ( Formenti *et al.*, 2022), BUSCO ( Manni *et al.*, 2021) and MERQURY.FK ( Rhie *et al.*, 2020).

The mitochondrial genome was assembled using MitoHiFi ( Uliano-Silva *et al.*, 2023), which runs MitoFinder ( Allio *et al.*, 2020) and uses these annotations to select the final mitochondrial contig and to ensure the general quality of the sequence.

***Assembly curation***

The assembly was decontaminated using the Assembly Screen for Cobionts and Contaminants (ASCC) pipeline (article in preparation). Flat files and maps used in curation were generated in TreeVal ( Pointon *et al.*, 2023). Manual curation was primarily conducted using PretextView ( Harry, 2022), with additional insights provided by JBrowse2 ( Diesh *et al.*, 2023) and HiGlass ( Kerpedjiev *et al.*, 2018). Scaffolds were visually inspected and corrected as described by Howe *et al*. (2021). Any identified contamination, missed joins, and mis-joins were corrected, and duplicate sequences were tagged and removed. The curation process is documented at https://gitlab.com/wtsi-grit/rapid-curation (article in preparation).

***Evaluation of the final assembly***

The final assembly was post-processed and evaluated with the three Nextflow ( Di Tommaso *et al.*, 2017) DSL2 pipelines “sanger-tol/readmapping” ( Surana *et al.*, 2023a), “sanger-tol/genomenote” ( Surana *et al.*, 2023b), and “sanger-tol/blobtoolkit” ( Muffato *et al.*, 2024). The pipeline sanger-tol/readmapping aligns the Hi-C reads with bwa-mem2 ( Vasimuddin *et al.*, 2019) and combines the alignment files with SAMtools ( Danecek *et al.*, 2021). The sanger-tol/genomenote pipeline transforms the Hi-C alignments into a contact map with BEDTools ( Quinlan & Hall, 2010) and the Cooler tool suite ( Abdennur & Mirny, 2020), which is then visualised with HiGlass ( Kerpedjiev *et al.*, 2018). It also provides statistics about the assembly with the NCBI datasets ( Sayers *et al.*, 2024) report, computes *k*-mer completeness and QV consensus quality values with FastK and MERQURY.FK, and a completeness assessment with BUSCO ( Manni *et al.*, 2021).

The sanger-tol/blobtoolkit pipeline is a Nextflow port of the previous Snakemake Blobtoolkit pipeline ( Challis *et al.*, 2020). It aligns the PacBio reads with SAMtools and minimap2 ( Li, 2018) and generates coverage tracks for regions of fixed size. In parallel, it queries the GoaT database ( Challis *et al.*, 2023) to identify all matching BUSCO lineages to run BUSCO ( Manni *et al.*, 2021). For the three domain-level BUSCO lineage, the pipeline aligns the BUSCO genes to the Uniprot Reference Proteomes database ( Bateman *et al.*, 2023) with DIAMOND ( Buchfink *et al.*, 2021) blastp. The genome is also split into chunks according to the density of the BUSCO genes from the closest taxonomically lineage, and each chunk is aligned to the Uniprot Reference Proteomes database with DIAMOND blastx. Genome sequences that have no hit are then chunked with seqtk and aligned to the NT database with blastn ( Altschul *et al.*, 1990). All those outputs are combined with the blobtools suite into a blobdir for visualisation.

The genome assembly and evaluation pipelines were developed using the nf-core tooling ( Ewels *et al.*, 2020), use MultiQC ( Ewels *et al.*, 2016), and make extensive use of the Conda package manager, the Bioconda initiative ( Grüning *et al.*, 2018), the Biocontainers infrastructure ( da Veiga Leprevost *et al.*, 2017), and the Docker ( Merkel, 2014) and Singularity ( Kurtzer *et al.*, 2017) containerisation solutions.

Table 4 contains a list of relevant software tool versions and sources.

**Table 4.** Software tools: versions and sources.

Software tool	Version	Source
BEDTools	2.30.0	https://github.com/arq5x/bedtools2
BLAST	2.14.0	ftp://ftp.ncbi.nlm.nih.gov/blast/executables/blast+/
BlobToolKit	4.3.7	https://github.com/blobtoolkit/blobtoolkit
BUSCO	5.4.3 and 5.5.0	https://gitlab.com/ezlab/busco
bwa-mem2	2.2.1	https://github.com/bwa-mem2/bwa-mem2
Cooler	0.8.11	https://github.com/open2c/cooler
DIAMOND	2.1.8	https://github.com/bbuchfink/diamond
fasta_windows	0.2.4	https://github.com/tolkit/fasta_windows
FastK	427104ea91c78c3b8b8b49f1a7d6bbeaa869ba1c	https://github.com/thegenemyers/FASTK
Gfastats	1.3.6	https://github.com/vgl-hub/gfastats
GoaT CLI	0.2.5	https://github.com/genomehubs/goat-cli
Hifiasm	0.19.8-r603	https://github.com/chhylp123/hifiasm
HiGlass	44086069ee7d4d3f6f3f0012569789ec138f42b84 aa44357826c0b6753eb28de	https://github.com/higlass/higlass
Merqury.FK	d00d98157618f4e8d1a9190026b19b471055b22e	https://github.com/thegenemyers/MERQURY.FK
MitoHiFi	3	https://github.com/marcelauliano/MitoHiFi
MultiQC	1.14, 1.17, and 1.18	https://github.com/MultiQC/MultiQC
NCBI Datasets	15.12.0	https://github.com/ncbi/datasets
Nextflow	23.04.0-5857	https://github.com/nextflow-io/nextflow
PretextView	0.2	https://github.com/sanger-tol/PretextView
purge_dups	1.2.5	https://github.com/dfguan/purge_dups
samtools	1.16.1, 1.17, and 1.18	https://github.com/samtools/samtools
sanger-tol/ascc	-	https://github.com/sanger-tol/ascc
sanger-tol/genomenote	1.1.1	https://github.com/sanger-tol/genomenote
sanger-tol/readmapping	1.2.1	https://github.com/sanger-tol/readmapping
Seqtk	1.3	https://github.com/lh3/seqtk
Singularity	3.9.0	https://github.com/sylabs/singularity
TreeVal	1.0.0	https://github.com/sanger-tol/treeval
YaHS	1.2a.2	https://github.com/c-zhou/yahs

### Wellcome Sanger Institute – Legal and Governance

The materials that have contributed to this genome note have been supplied by a Darwin Tree of Life Partner. The submission of materials by a Darwin Tree of Life Partner is subject to the **‘Darwin Tree of Life Project Sampling Code of Practice’**, which can be found in full on the Darwin Tree of Life website here. By agreeing with and signing up to the Sampling Code of Practice, the Darwin Tree of Life Partner agrees they will meet the legal and ethical requirements and standards set out within this document in respect of all samples acquired for, and supplied to, the Darwin Tree of Life Project. 

Further, the Wellcome Sanger Institute employs a process whereby due diligence is carried out proportionate to the nature of the materials themselves, and the circumstances under which they have been/are to be collected and provided for use. The purpose of this is to address and mitigate any potential legal and/or ethical implications of receipt and use of the materials as part of the research project, and to ensure that in doing so we align with best practice wherever possible. The overarching areas of consideration are:

•   Ethical review of provenance and sourcing of the material

•   Legality of collection, transfer and use (national and international) 

Each transfer of samples is further undertaken according to a Research Collaboration Agreement or Material Transfer Agreement entered into by the Darwin Tree of Life Partner, Genome Research Limited (operating as the Wellcome Sanger Institute), and in some circumstances other Darwin Tree of Life collaborators.

## Data Availability

European Nucleotide Archive: Conger conger (European conger). Accession number PRJEB65259; https://identifiers.org/ena.embl/PRJEB65259 ( Wellcome Sanger Institute, 2023). The genome sequence is released openly for reuse. The *Conger conger* genome sequencing initiative is part of the Darwin Tree of Life (DToL) project. All raw sequence data and the assembly have been deposited in INSDC databases. The genome will be annotated using available RNA-Seq data and presented through the Ensembl pipeline at the European Bioinformatics Institute. Raw data and assembly accession identifiers are reported in Table 1 and Table 2.

## Author information

Members of the Marine Biological Association Genome Acquisition Lab are listed here: https://doi.org/10.5281/zenodo.8382513.

Members of the Darwin Tree of Life Barcoding collective are listed here: https://doi.org/10.5281/zenodo.12158331

Members of the Wellcome Sanger Institute Tree of Life Management, Samples and Laboratory team are listed here: https://doi.org/10.5281/zenodo.12162482.

Members of Wellcome Sanger Institute Scientific Operations: Sequencing Operations are listed here: https://doi.org/10.5281/zenodo.12165051.

Members of the Wellcome Sanger Institute Tree of Life Core Informatics team are listed here: https://doi.org/10.5281/zenodo.12160324.

Members of the Tree of Life Core Informatics collective are listed here: https://doi.org/10.5281/zenodo.12205391.

Members of the Darwin Tree of Life Consortium are listed here: https://doi.org/10.5281/zenodo.4783558.
