# Supplementary material for: The genome sequence of the European conger eel, Conger conger (Linnaeus, 1758)
Source: Wellcome Open Res. 2024 Sep 18;9:532. [Version 1] doi: 10.12688/wellcomeopenres.23052.1 (PMC11480707; doi:10.12688/wellcomeopenres.23052.1)
Supplement: Peer review history

# Reviewer report: Reviewer response for version 1

**DOI:** 10.21956/wellcomeopenres.25388.r101469

**Published:** 2024-10-15

**Authors:** Kazuharu Nomura

**Affiliations:** 1 Fisheries Technology Institute, Japan Fisheries Research and Education Agency, Mie, Japan

This manuscript is a Data Note type of submission that provides a chromosome-level genome assembly of the European conger eel ( *Conger conger*). The paper is overall very clear, with detailed descriptions of the pipelines used to generate the dataset. The protocols are appropriate and technically sound, and the accuracy of the resulting genome assembly is reasonable according to current standards. All data and tools have been made publicly available, allowing other researchers easy access to this valuable resource. The overall quality of the research is high, and the provided data will be useful for future studies in the conservation and management of this species. Therefore, I would recommend that this paper be considered for indexing in Wellcome Open Research.

Are sufficient details of methods and materials provided to allow replication by others?

Yes

Is the rationale for creating the dataset(s) clearly described?

Yes

Are the datasets clearly presented in a useable and accessible format?

Yes

Are the protocols appropriate and is the work technically sound?

Yes

Reviewer Expertise:

Genetics

I confirm that I have read this submission and believe that I have an appropriate level of expertise to confirm that it is of an acceptable scientific standard.

# Reviewer report: Reviewer response for version 1

**DOI:** 10.21956/wellcomeopenres.25388.r101468

**Published:** 2024-10-15

**Authors:** Khairul Syahputra

**Affiliations:** 1 Research Center for Fishery, National Research and Innovation Agency (BRIN), Cibinong, Indonesia

**Comments on manuscript of European conger genome:**

 The present study is an interesting work providing genome sequence of European conger. It can be useful for further studies related to genome references in Eel and other aquatic species. However, some minor issues exist in the text. They should be addressed before the manuscript is suitable for indexing.

**Title:**

 “eel” is not necessary and can be omitted!

**Abstract:**

 Common name of species should come first before its scientific name. I do not think that the classification of species deserves to be stated here! We can find it on the Species Taxonomy section!

**Background:**

 As suggested previously, “eel” on common name of species is not necessarily included.

 Line 5 – 500 m???

 7 – “eel” or Eels”??

 11 -2.7 m in length???

 Second paragraph – Justification of using European eel as fish model in this study deserves more explanation here and why this species is focused on the present study!

**Genome sequence report:**

 Line 1 – use a common name to mention the species. The scientific name is only included at the first appearance of species in the text. Apply it thorough manuscript!

 Paragraph 2 line 3 – “1,009 gaps” is not found in Table2!

**Methods:**

*Sample acquisition and barcoding*

 First paragraph, last sentence – a) What were tissue samples collected, and b) How long were the tissue sample kept on dry ice before nucleic acid extraction? State this!

 2 ^nd^ Paragraph line 3 – What tissue? Is there any specific size or weight?

*Library preparation and Sequencing:*

 Why did the authors use DNA and RNA to build the genome sequence of European eel? Explain this in the text!

Are sufficient details of methods and materials provided to allow replication by others?

Partly

Is the rationale for creating the dataset(s) clearly described?

Yes

Are the datasets clearly presented in a useable and accessible format?

Yes

Are the protocols appropriate and is the work technically sound?

Yes

Reviewer Expertise:

Fish Genetic

I confirm that I have read this submission and believe that I have an appropriate level of expertise to confirm that it is of an acceptable scientific standard.

# Reviewer report: Reviewer response for version 1

**DOI:** 10.21956/wellcomeopenres.25388.r100177

**Published:** 2024-09-23

**Authors:** Chong Han

**Affiliations:** 1 Guangzhou University, Guangzhou, Guangdong, China

This study completed a chromosome-level assembly of European conger eel based on pacbio and Hi-C technology. It is a good work with detailed methods. All the protocols were appropriate and technically sound. Besides, we could find the related data in the NCBI database. In all, it is a valuable research, which is worthy to be Indexed in Wellcome Open Research.

Are sufficient details of methods and materials provided to allow replication by others?

Yes

Is the rationale for creating the dataset(s) clearly described?

Yes

Are the datasets clearly presented in a useable and accessible format?

Yes

Are the protocols appropriate and is the work technically sound?

Yes

Reviewer Expertise:

endocrine disturbs, genome assembly and transcriptome

I confirm that I have read this submission and believe that I have an appropriate level of expertise to confirm that it is of an acceptable scientific standard.
